# Correlation of the anatomical sacral slope with pelvic incidence in female patients with developmental hip dysplasia: a retrospective cross-sectional study

**DOI:** 10.1186/s13018-020-02022-9

**Published:** 2020-10-21

**Authors:** Norio Imai, Hayato Suzuki, Atsushi Sakagami, Yuki Hirano, Naoto Endo

**Affiliations:** 1 Division of Comprehensive Musculoskeletal Medicine, Niigata University Graduate School of Medical and Dental Sciences, 1-757, Asahimachi-dori, Chuo ku, Niigata City, Niigata Prefecture 951-8510 Japan; 2 Division of Orthopedic Surgery, Department of Regenerative and Transplant Medicine, Niigata University Graduate School of Medical and Dental Sciences, 1-757, Asahimachi-dori, Chuo ku, Niigata City, Niigata Prefecture 951-8510 Japan

**Keywords:** Anatomical sacral slope, Developmental dysplasia of the hip, Lumbar lordosis, Pelvic incidence

## Abstract

**Background:**

The anatomical sacral slope is considered as an anatomical pelvic parameter independent of femoral head centers for measurement of anatomical sacral slope and was previously described to strongly correlate with pelvic incidence on a two-dimensional examination of healthy subjects. However, the correlation between anatomical sacral slope and pelvic incidence was unclear in patients with developmental dysplasia of the hip. This study aimed to examine the correlation between anatomical sacral slope and other spinopelvic parameters by analyzing plain radiographs of female patients with developmental dysplasia of the hip.

**Methods:**

Eighty-four women with developmental dysplasia of the hip were examined. Lumbar lordosis, thoracic kyphosis, pelvic incidence, sacral slope, and anatomical sacral slope (the angle formed by the straight line of the S1 superior endplate and a line at a right angle to the anterior pelvic plane) were determined by analyzing plain radiographs. The correlations were examined by Pearson’s correlation coefficients, and intra- and inter-rater intraclass correlation coefficients were evaluated for reliability.

**Results:**

A strong correlation was observed between pelvic incidence and anatomical sacral slope (*r =* 0.725, *p* < 0.001). In addition, the correlation between anatomical sacral slope and lumbar lordosis was similar to that between pelvic incidence and lumbar lordosis (*r =* 0.661, *p* < 0.001, and *r =* 0.554, *p* < 0.001, respectively). The intra-rater intraclass correlation coefficient values were 0.869 and 0.824 for anatomical sacral slope and pelvic incidence, respectively. Furthermore, the inter-rater intraclass correlation coefficient values were 0.83 and 0.685 for anatomical sacral slope and pelvic incidence, respectively.

**Conclusions:**

We observed that the strong correlation between anatomical sacral slope and pelvic incidence in patients with developmental dysplasia of the hip was equal to that in normal healthy subjects. The correlation between anatomical sacral slope and lumbar lordosis was equal to that between pelvic incidence and lumbar lordosis. Additionally, the intraclass correlation coefficient values for the anatomical sacral slope were slightly higher than those for pelvic incidence. Thus, we conclude that anatomical sacral slope can be considered as a helpful anatomical pelvic parameter that is a substitute for pelvic incidence not only in normal healthy subjects, but also in patients with developmental dysplasia of the hip.

## Background

Based on previous studies, it was considered that pelvic morphology, as well as pelvic incidence (PI), influences sagittal spinal alignments, such as lumbar lordosis (LL), sacral slope (SS) [1–3], and standing posture [4–6]. A larger PI is considered to be a risk factor for spondylolisthesis because it seemingly leads to anterior deviation of the sagittal vertical axis [7, 8]. Additionally, the discrepancy between PI and LL leads to spinal deformity in adults [9]. In sagittal spinal malalignment, maintaining a suitable balance is considered difficult, and it may lead to “hip-spine syndrome” [10]. Consequently, PI is considered as one of the most important clinical parameters and should be evaluated.

Generally, many surgeons evaluate sagittal thoracolumbar spinal alignment and pelvic parameters by analyzing two-dimensional (2D) plain radiographs captured in the standing position [11, 12]. SS and pelvic tilt (PT) are defined as functional parameters, since these angles are influenced by the anteroposterior tilt of the pelvis, that is, anterior or posterior tilt in the sagittal plane in the standing position. On the contrary, PI is deemed to be an anatomical parameter as it is not influenced by the anteroposterior tilt of the pelvis. SS and PT are related to PI in geometrical relation by the formula PI = SS + PT.

We recently described a correlation between PI and anatomical sacral slope (a-SS), SS relative to anterior pelvic plane (APP), in normal healthy subjects using 2D and three-dimensional (3D) measurements [1, 13, 14]. The a-SS was considered as an anatomical parameter that does not require femoral head measurements, as is the case when determining the PI. This is advantageous as the femoral head center is sometimes difficult to establish. Previously, we observed a close correlation between PI and a-SS among normal healthy subjects and patients with developmental dysplasia of the hip (DDH) measured using only the 3D method [13]. However, the correlation between PI and a-SS has not been examined in patients with DDH, wherein pelvic and/or spinal morphological features observed using 2D measurements may be different from those of normal subjects.

This study aimed to examine the correlation between PI and a-SS using plain radiographs of patients with DDH. Similarly, we examined the correlation between a-SS and LL using 2D measurements.

## Methods

Eighty-four women with bilateral DDH, who had undergone curved periacetabular osteotomy [15] for treating early-stage hip osteoarthritis due to DDH from April 1, 2010, to July 30, 2017, were examined in our hospital. The inclusion criterion was a center-edge angle of the hip joints less than 20°, obtained from the anteroposterior view of the hips on plain radiographs. This is because these patients seemed to have a common morphological characteristic of DDH in their pelvis and might have a common functional alignment in the pelvis and spine. We excluded subjects who had undergone any hip joint surgery, those who were evaluated to have hip dysplasia of Crowe stages 2–4 [16] regarding subluxation, or those in which arthritic change was evaluated as Tonnis grades 2–3 [17] observed on plain radiographs of the hip.

The Ethical Review Board of our institution approved this study and waived the need for informed consent because of the retrospective cross-sectional design of the study.

### Measurements of pelvic and thoracolumbar parameters

**Fig. 1:**
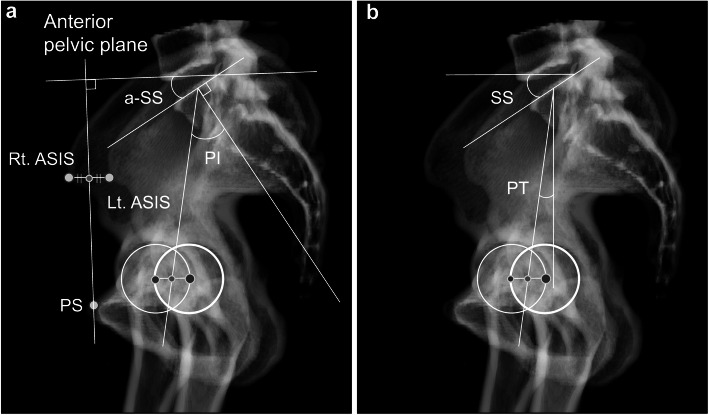
Anatomical and functional parameters of the pelvis. **a** Anatomical parameters. **b** functional parameters. PI, pelvic incidence; SS, sacral slope; APP, anterior pelvic plane; L- and R-ASIS, left and right anterior superior iliac spine

**Fig. 2:**
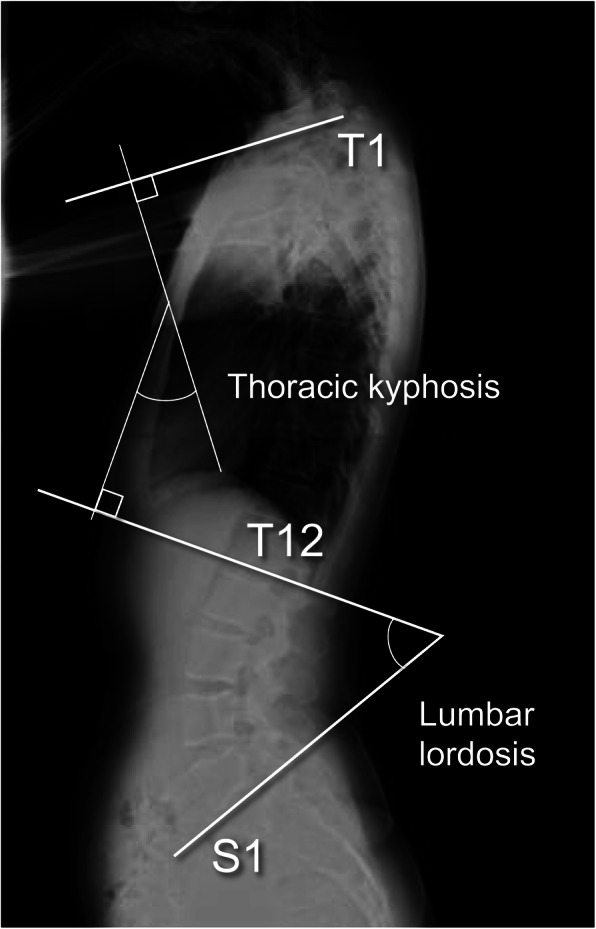
Sagittal thoracolumbar spinal parameters. Lumbar lordosis (LL) was established by the T12 inferior end plate and the S1 superior end plate. Thoracic kyphosis (TK) is measured between the T1 superior end plate and the T12 inferior end plate

The pelvic parameters, such as PI, SS, and a-SS, and the thoracolumbar parameters, such as thoracic kyphosis (TK) and LL, were measured using thoracic and lumbar plain radiographs including the pelvis in the standing position. PI was established as the angle formed by the line at a right angle to the superior endplate of S1 at its middle point and the line connecting this point to the axis linking the bilateral femoral heads (Fig. 1) [11]. The SS was established as the angle formed by the straight line of the S1 superior endplate and a leveled line at a right angle to the gravitational force direction. PT was established as the angle formed by the straight line connecting the middle point of the S1 endplate to the hip axis and the vertical line parallel to the direction of gravitational force (Fig. 1). Further, LL was established as the angle from the line of T12 inferior endplate and the line of the S1 superior endplate (Fig. 2). TK was defined as the angle formed by the line of the T1 superior endplate and the line of the T12 inferior endplate (Fig. 2).

### Statistical analysis

We analyzed the data using SPSS software (version 24; SPSS, Inc., Chicago, IL). The correlation of PI, SS, a-SS, LL, and TK were evaluated with Pearson’s correlation coefficients according to Guilford’s definition [18]. Similarly, we evaluated the statistical power (type II (β) error) using a post hoc analysis, with 0.3 as the effect size (*d*) and 0.05 as type I (*α*) error, for the correlation analysis. We evaluated the validity of this study by calculating the mean absolute difference (MAD), the variability by the standard deviation (SD), and the intra- and inter-rater reliabilities with interclass correlation coefficients (ICCs) (with 95% confidence intervals) by two-tailed analysis. We measured 1-week intervals twice to determine the intra-rater reliability and drew a parallel between the measurements examined by two other observers to assess the inter-rater reliability. Values of *p* below 0.05 were considered statistically significant.

## Results

The average age and body mass index of the participants were 35.0 ± 9.2 years (range 20–52 years) and 22.0 ± 2.9 kg/m^2^ (range 16.2–27.8 kg/m^2^), respectively.

**Fig. 3:**
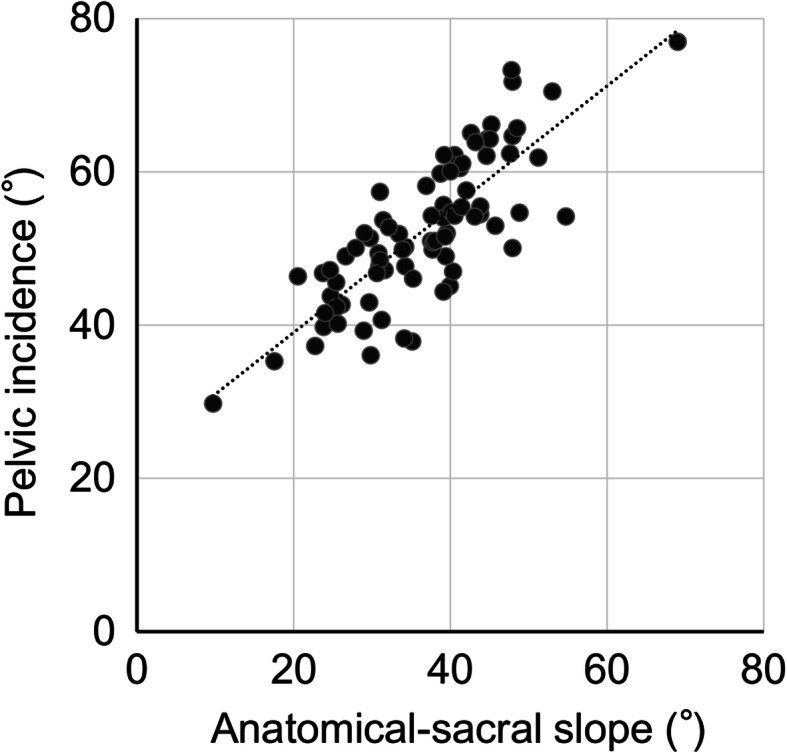
The relationship between pelvic incidence and anatomical-sacral slope. Pelvic incidence was strongly related to anatomical-sacral slope

Table 1 shows the details of the parameters. A close correlation was observed between PI and a-SS (*r* = 0.725, *p* < 0.001) (Fig. 3), as defined by Guilford [18] (Table 2). The regression formula calculated from this correlation is as follows: PI = 0.8 × a-SS + 21.

**Table 1:** The details of spinopelvic and spinal parameters of the 84 patients with developmental dysplasia of the hip

PI	54.2 ± 10.6 (31.0–77.0°)
SS	38.5 ± 10.8 (10.0–69.0°)
PT	15.7 ± 7.0 (− 8.0 to 27.0°)
a-SS	40.8 ± 9.4 (20.0–61.0°)
TK	35.0 ± 10.7 (7.0–83°)
LL	55.4 ± 18.4 (3.0–83.0°)

Mean ± standard deviation (range)

*PI* Pelvic incidence, *SS* Sacral slope, *PT* Pelvic tilt, *a-SS* Anatomical sacral slope, *TK* Thoracic kyphosis, *LL* Lumbar lordosis

**Table 2:** Pearson’s correlation coefficients of pelvic and sagittal spinal parameters

	SS	PT	a-SS	LL	TK
PI	0.632*	0.341*	0.725*	0.554*	− 0.017
SS		− 0.229	0.698*	0.827*	0.141
PT			0.128	0.034	− 0.068
a-SS				0.661*	0.057

*PI* Pelvic incidence, *SS* Sacral slope, *PT* Pelvic tilt, *a-SS* Anatomical sacral slope, *TK* Thoracic kyphosis, *LL* Lumbar lordosis

*p < 0.05

Regarding the correlation between pelvic and thoracolumbar parameters, a strong correlation was observed between SS and LL (*r* = 0.827, *p* < 0.001). Concerning the anatomical parameters, the correlation between a-SS and LL was equal to that between PI and LL (*r* = 0.554, *p* < 0.001, and *r* = 0.661, *p* < 0.001, respectively) (Table 2). However, no correlation was observed between TK and PI, SS, or a-SS. The power analysis of the correlation showed a power value of 0.803. Intra-rater MADs ranged from 2.6° for SS to 3.7° for PI, and the smallest ICC was 0.708 for TK (Table 3). As regards the MADs, intra-rater MADs were slightly smaller than the inter-rater MADs (the largest MAD was 4.5° for PI), and the smallest ICC was 0.685 for PI (Table 3).

**Table 3:** Intra- and interrater reliabilities of the measured values

	Intra-rater reliability	Inter-rater reliability
PI	3.7 ± 2.8° (0.824*)	4.5 ± 3.6° (0.685*)
SS	2.6 ± 2.2° (0.869*)	3.5 ± 2.8° (0.712*)
PT	2.9 ± 2.8° (0.842*)	4.0 ± 3.2° (0.697*)
a-SS	2.9 ± 2.6° (0.868*)	3.7 ± 2.7° (0.835*)
TK	3.4 ± 3.0° (0.708*)	3.9 ± 3.8° (0.698*)
LL	3.0 ± 2.4° (0.823*)	3.6 ± 3.8° (0.714*)

Mean absolute difference ± standard deviation (intraclass correlation coefficient)

*PI* Pelvic incidence, *SS* Sacral slope, *PT* Pelvic tilt, *a-SS* Anatomical sacral slope, *TK* Thoracic kyphosis, *LL* Lumbar lordosis

*p < 0.05

## Discussion

In this study, a strong correlation was observed between PI and a-SS; consequently, PI was considered feasible of being estimated from a-SS. Moreover, the correlation between a-SS and LL was similar to that between PI and LL (*r* = 0.661, *p* < 0.001, and *r* = 0.554, *p* < 0.001, respectively). These results were similar to those of a study in patients with DDH using 3D measurements [13] and results in normal healthy subjects, obtained by 2D measurements [1]. Consequently, the relationships between a-SS and LL and between PI and LL were similar among patients with DDH and normal subjects. The mean values in patients with DDH were as follows: PI, 54.2°; SS, 38.5°; and LL, 55.43°. In normal adults, the measured values of PI, SS, and LL have been described to range between 44.6 and 57.7°, 32.5 and 41.4°, and 48.2 and 57.2°, respectively [19, 20]. Formerly, PI had been reported to be strongly correlated to SS and also to LL in normal women [8]. Our findings were similar to those of previous results [13]. Therefore, the results of our study can be considered valid.

PI-LL discrepancy (PI-LL ≥ 11°) has been described to likely lead to disability in patients with spinal deformities [9]. Following spinal fusion surgery for lumbar degenerative diseases, PI-LL discrepancy reportedly leads to residual symptoms, such as lumbago and other disabilities [21, 22]. Therefore, measuring the exact PI is essential. However, measuring PI requires identifying femoral head centers, which is sometimes arduous, especially in patients with aspherical femoral heads and with subluxation following osteoarthritis of the hip. In these patients, a new parameter, independent of the femoral head, seems to be required. Moreover, placing the acetabular component within a moderate level might be required to avoid increasing PI after total hip arthroplasty.

In this study, we observed that PI was strongly correlated to a-SS on 2D radiologic measurements. These findings were similar to those of several previous studies that described a strong correlation between PI and a-SS on 3D measurements in patients with DDH [13]. From these results, PI could be estimated from a-SS using the following regression formula: PI = 0.8 × a-SS + 21. Moreover, this formula is similar to that used in normal healthy subjects: PI = 0.8 × a-SS + 18 [1].

Additionally, the findings showing a significant correlation between a-SS and LL were similar to those for PI and LL in patients with DDH and normal healthy subjects [13]. Therefore, a-SS may be useful to estimate PI in normal healthy subjects and patients with DDH and may be considered as a new anatomical pelvic parameter that is independent of the femoral head center for measurements. This is because measurements involving the femoral head were occasionally unsuitable, such as in patients with aspherical, flattening, or dislocated femoral heads.

Additionally, intra-rater and inter-rater MADs of PI, which included the femoral head center for measurements, were larger than those of a-SS, equal to those in normal subjects [13]. Therefore, a-SS may lead to higher reliability than PI when using 2D radiological measurements.

This study had several limitations. First, the sample size was small; however, the power value in the correlation analysis was 0.803; therefore, the sample size of this study was considered sufficient by power analysis. Second, this study only included Japanese individuals as participants. Previously, differences in sagittal thoracolumbar spinal and pelvic parameters among races had been described [23]. Therefore, our findings may be different from those in other races. Further studies are needed to investigate the generalizability of this result to other populations. Third, only female patients were included. DDH is predominant in females, with a female to male ratio of 9:1 [24]. Furthermore, < 20 male patients have undergone periacetabular osteotomy during the last 10 years in our hospital. Finally, we exclusively evaluated Crowe type 1 hip dysplasia; however, since the number of patients with high dislocation was less common, the influence seems not to be significant.

## Conclusions

Anatomical sacral slope (a-SS), a novel parameter, can be considered convenient and can be examined by 2D plain radiographs. Additionally, the correlation between a-SS and LL was similar to that between PI and LL, and the 2D and 3D findings were similar in patients with DDH [13]. Thus, we believe that a-SS, which does not require the femoral head center for measurement, is a useful and a new suggested anatomical pelvic parameter that may be available instead of PI. Further large-scale studies are required to evaluate the validity and usefulness of a-SS as an anatomical parameter.

## Data Availability

All data generated or analyzed during this study are included in this published article.

## Abbreviations

2D: Two-dimensional
3D: Three-dimensional
APP: Anterior pelvic plane
a-SS: Anatomical sacral slope
DDH: Developmental dysplasia of the hip
ICCs: Interclass correlation coefficients
LL: Lumbar lordosis
MAD: Mean absolute difference
PI: Pelvic incidence
PT: Pelvic tilt
SS: Sacral slope
TK: Thoracic kyphosis
